# A Comparison of Miniature Lattice Structures Produced by Material Extrusion and Vat Photopolymerization Additive Manufacturing

**DOI:** 10.3390/polym13132163

**Published:** 2021-06-30

**Authors:** Rafael Guerra Silva, María Josefina Torres, Jorge Zahr Viñuela

**Affiliations:** School of Mechanical Engineering, Pontificia Universidad Católica de Valparaíso, Av. Los Carrera 01567, Quilpué 2430000, Chile; josefina.torres@pucv.cl (M.J.T.); jorge.zahr@pucv.cl (J.Z.V.)

**Keywords:** lattices, cellular materials, additive manufacturing, material extrusion, vat photopolymerization, digital light processing, compressive behavior, energy absorption

## Abstract

In this paper, we study the capabilities of two additive manufacturing technologies for the production of lattice structures, namely material extrusion and vat photopolymerization additive manufacturing. A set of polymer lattice structures with diverse unit cell types were built using these additive manufacturing methods and tested under compression. Lattice structures built using material extrusion had lower accuracy and a lower relative density caused by the air gaps between layers, but had higher elastic moduli and larger energy absorption capacities, as a consequence of both the thicker struts and the relatively larger strength of the feedstock material. Additionally, the deformation process in lattices was analyzed using sequential photographs taken during the compression tests, evidencing larger differences according to the manufacturing process and unit-cell type. Both additive manufacturing methods produced miniature lattice structures with similar mechanical properties, but vat polymerization should be the preferred option when high geometrical accuracy is required. Nevertheless, as the solid material determines the compressive response of the lattice structure, the broader availability of feedstock materials gives an advantage to material extrusion in applications requiring stiffer structures or with higher energy absorption capabilities.

## 1. Introduction

Since its inception in the 1980s, additive manufacturing (AM) had become a key element in engineering, enabling the fast development of parts with complex geometries [1,2]. AM is considered an excellent alternative for producing lightweight, geometrically complex parts such as lattices [3], as it offers control over cell size and shape, shape and size of struts, the topology of the structure, and many other features [4]. Lattice structures have applications in various fields such as medical, aeronautical, and automobile industries for their weight reduction and high energy absorption capabilities [5,6,7,8].

During the last decade, special attention has been given to the development of design and optimization methods for lattice structures [6,9,10,11,12]. Moreover, the fabrication and testing for a wide variety of lattices fabricated using different AM processes have been reported, including material extrusion (ME) [13,14,15,16,17], vat photopolymerization (VP) [18,19,20,21,22], jet fusion (JF) [23,24,25], and selective laser sintering (SLS) [21,26,27,28].

In recent years, two AM technologies have enabled the development of several polymer-based consumer-grade 3D printers at low costs, ME and digital light processing (DLP). ME is an additive manufacturing process, in which a thermoplastic filament is driven into an extruder head where it is melted through a nozzle to produce a part layer by layer [29]. ME is capable of creating parts with an accuracy in the range of 0.1–0.6 mm using polymers with relatively low melting temperatures such as acrylonitrile butadiene styrene (ABS) and polylactic acid (PLA), as feedstock material [30,31]. ME advantages include ease of use, low cost and wide availability of feedstock materials and equipment.

DLP is a variant of the vat photopolymerization process, a group of AM processes that also include stereolithography (SLA) [1]. VP uses a photopolymer as feedstock material, which is stored in a vat and treated with either visible or UV light. The curing light triggers the polymerization reaction, turning the liquid resin into a solid part. In contrast to SLA, in DLP a digital light projector is employed instead of a laser source and a reflecting mirror [1]. DLP is capable of fabricating with high accuracy (0.025–0.1 mm) with smooth surfaces, although the number of photocurable materials is more limited [1,32]. Additionally, they are known to degrade over time, resulting in poorer mechanical properties [1].

Although ME could seem inadequate for the production of 3D miniature lattice structures, the feedstock is safer and easier to handle and does not require additional post-processing. Previous authors have studied the fabrication of miniature polymer lattice structures using ME. Al Rifaie et al. [15] studied the compression behavior of four variants of the cubic cell (cell size 5 mm, strut thickness 1 mm). Similarly, Karamooz Ravari et al. [13] tested the mechanical properties of ME-fabricated body-centered cubic lattices (strut diameter 1.5 mm). Other studies have evaluated the compressive response and energy absorption capacity of a handful of ME-fabricated lattices, but limiting their analysis to large unit cell size (>10 mm) [14,16]. Additionally, a comparison of the mechanical properties of lattices fabricated built by ME and multi-jet fusion (MJF), reporting that the MJF-fabricated samples had better properties than samples built using ME [33], although the study focused on lattices with large unit cell size (>10 mm).

In general, SLA/DLP processes are considered faster, more economical and capable of generating a variety of highly complex 3D structures with high precision [2,6,34]. Furthermore, support structures are not needed to build cellular and hollow structures [6]. Therefore, they are ideal for printing intricate parts, such as lattice meta-materials with high accuracy. However, there are very few studies using DLP, as the technology has been made available only recently.

Ling et al. [19] compared octet-truss lattice structures built by SLA using two polymer resins of different densities and strengths. The mechanical behavior of the structures was examined under both quasi-static and dynamic compressive loading. They reported that the mechanical response of the octet lattices depended on both the relative density and the intrinsic material properties. Additionally, higher density structures showed larger effective yield and compressive strength, while the basic printing material fundamentally determined its macroscopic properties: one material provided a brittle mechanical response under compression while the other provided a tough response.

Luxner et al. [21] compared the mechanical response of lattices with different unit cells (simple cubic, body-centered cubic and Gibson–Ashby) fabricated using both DLP and SLS under compression. The DLP lattice structures were built in resin (a blend of acrylates and epoxy-based, E = 2300 MPa), while the SLS structures were fabricated in polyamide (E = 2400 MPa). A comparison of the normalized elastic modulus in samples with a relative density of 0.1 revealed that the fabrication process had no effect in body-centered cubic lattices, but had a significant effect in simple cubic lattices. However, no details about either cell geometry or manufacturing parameters were explicitly given.

Zhou [35] reported on the manufacturability of selected miniature lattice structures using different AM technologies, including material extrusion, selective laser sintering, stereolithography and direct light processing, reporting that ME and SLS did not have an adequate resolution, in opposition to SLA and DLP. On the other hand, the high cost of SLA and resin degradation in DLP were identified as the main challenges. No mechanical testing of the AM-fabricated lattices was carried out. Afterward, Guerra Silva et al. [17] evaluated the capabilities of ME using the same benchmark lattices, demonstrating that it is possible to produce 3D miniature lattice structures with complex topologies using ME, even if accuracy was still low.

Further information on AM and testing of lattices is available in the extended summary presented by Dong et al. [36]. Uribe-Lam et al. [34] also reviewed the different AM technologies used to build lattices, comparing their capabilities and limitations, and proposing general guidelines for their use.

Studies comparing AM methods have been carried out also for solid parts with larger dimensions. Shah et al. [37] compared three different AM methods, identifying several limitations in the comparison methods. In that study, samples of a hollow cylindrical benchmarking artifact were built using ME, SLA and SLS, and measured using computed tomography and a coordinate-measuring machine (CMM). In terms of the accuracy of AM methods, the ME sample had the worst performance. However, SLA showed issues with internal structures, and SLS was the most difficult to measure using CMM, due to small powder deposits formed after each measuring. Further studies comparing SLA and ME have identified criteria for the selection of either method [38]: ME can produce models with different percentages of infill, enabling a significant reduction in weight, material use, time and cost of production; on the other hand, SLA offers high accuracy, higher speed, smoother surfaces and transparency. Additional advantages of ME include the capability of producing multi-material parts and the availability of recycled feedstock material [39].

Although some studies have compared the performance of parts produced by different AM processes, even considering the fabrication of lattice structures, a comprehensive comparison of ME- and DLP-fabricated lattices is yet to be reported in the literature. Given the increasing availability of both technologies and the growing interest in the fabrication of miniature lattices [40,41], it would be of interest to assess the mechanical properties and characteristics of these structures using both AM methods.

## 2. Materials and Methods

A set of eight lattice structures proposed by Zhou [35] was used as a benchmark to compare the two AM processes, all designed using the software nTopology [42]. The benchmark of lattice structures evaluated in both studies is available online [43]. A detailed analysis of the geometric features of the eight lattice structures (dimensions, cell size, strut diameter, strut orientation) is presented in Table 1.

### 2.1. Specimen Manufacturing

ME test specimens were prepared using a desktop machine (model Up mini 2, Tiertime, Beijing, China), and white ABS (Tiertime) was used as feedstock material. The digital models (STL files) were processed using the slicing software Up Studio v2.6 (Tiertime). The same manufacturing parameters were used in the production of all samples: extruder temperature 270 °C, bed temperature 90 °C, layer height 0.15 mm, extrusion width 0.35 mm, nozzle diameter 0.4 mm. No support was used in the production of the samples, and a raft was used to secure surface adhesion to the bed. All ME samples were fabricated using the nominal 100% infill rate.

VP test specimens were prepared using a desktop machine (model LD-001, Creality, Shenzhen, China). The equipment uses a digital light projector as the source to cure the photosensitive resin. The slicing software 3D Creator Slicer (Creality) was used to process the STL files. Grey photosensitive epoxy resin (Creality) was used as feedstock materials. The manufacturing parameters, which are more limited in VP, remained unchanged in the production of the samples: layer thickness was set to 0.1 mm and the exposure time was set to 17 s. A longer exposure time was set for the first layer (80 s), as suggested by the equipment manufacturer. These parameters enabled the production of error-free, geometrically accurate lattices. The DLP-fabricated samples were rinsed in isopropyl alcohol, to remove the remaining liquid layer of resin. Afterward, these models were exposed to indirect natural sunlight for 24 h.

In both AM processes, support was not required during the manufacturing process, as the selected lattice structures were self-supporting. Similarly, no special methods were required to secure adhesion to the printing bed nor to remove the specimens from it.

The information available about the chemical composition and other properties of both feedstock materials is very limited (Table 2), so compression tests of solid samples were performed for printed samples of both feedstock materials fabricated using the corresponding AM process.

### 2.2. Compression Tests

A computerized electronic universal testing machine (model WDW-200E, TIME Group Inc., Beijing, China) with flat plates (diameter 200 mm) was used to carry out the compression tests. Specimens were tested in the same orientation of printing.

The compression tests of solid samples for both feedstock materials were performed under standard ASTM D695 [47]. The preparation of the lattice specimens and the compressive tests were carried out according to the standard test method for compressive properties of rigid cellular plastics, ASTM D1621 [48]. A constant speed of 2.3 mm/min was used during the compression tests. No lubricant was used in the contact surfaces between specimens and plates. Compression tests were carried out until the samples were compressed to about 50% strain, as the densification phase was beyond the scope of this study. For error estimation, two specimens of every configuration were manufactured and tested, totaling 32 samples.

Elastic modulus and energy absorption capacity for all samples were determined from the experimental data. The energy absorption capacity quantified was measured as the area under the stress-strain curve up to a value of 40% nominal strain.

## 3. Results

Mechanical properties of both feedstock materials (ABS and photopolymerized resin) obtained from the compression tests are presented in Table 3, evidencing some discrepancies between mechanical properties reported in the literature (Table 2) and the effective properties using the available consumer desktop machines.

**Figure 1:**
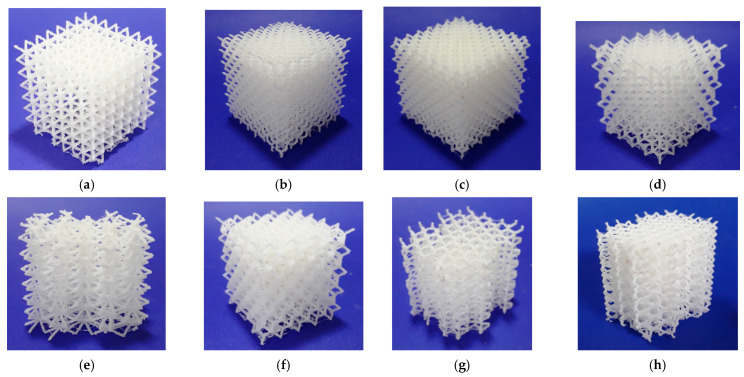
Lattice structures (built by ME): (**a**) CVC; (**b**) CD; (**c**) CF; (**d**) TV; (**e**) HPV; (**f**) TOV; (**g**) HPD; (**h**) HPL. Images licensed under the Creative Commons License [17].

**Figure 2:**
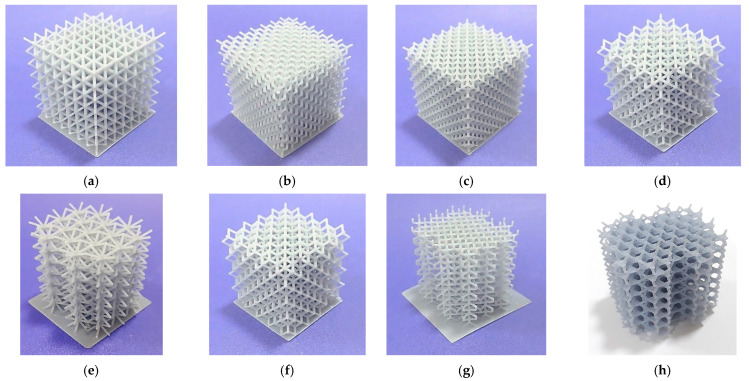
Lattice structures (built by DLP): (**a**) CVC; (**b**) CD; (**c**) CF; (**d**) TV; (**e**) HPV; (**f**) TOV; (**g**) HPD; (**h**) HPL.

The lattice structures fabricated using ME and VP are presented in Figure 1 and Figure 2 respectively.

**Figure 3:**
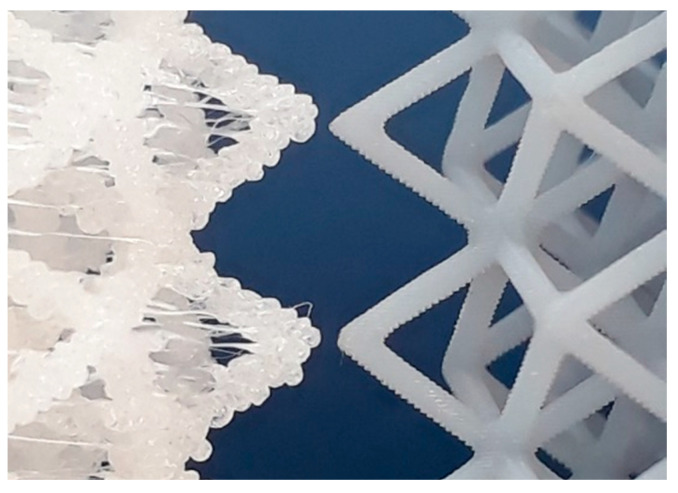
Side by side comparison of struts produced by material extrusion (**left**) and vat polymerization (**right**). In both lattices, the effect of stair-stepping is noticeable, with a stronger effect in struts fabricated by ME.

Both AM processes were capable of producing the lattices, although ME had lower productivity. The fabrication of a single sample using ME took up to 4 h, while the simultaneous production of up to four VP lattices required less than 2 h. Figure 3 presents two CVC lattices produced by ME and VP side by side. The effect of stair-stepping is noticeable in both lattices, although it is stronger in struts fabricated by ME. Both geometric and dimensional accuracy of ME struts were poorer. For instance, measured strut thickness in CVC lattices built by ME was 0.96 ± 0.08 mm, while in CVC lattices built by VP, it was 0.74 ± 0.04 mm, with the latter being closer to the digital STL model (0.70 mm).

Mean values and standard deviations (SD) for the relative density of the eight lattice configurations built by ME and VP are presented in Table 4.

**Figure 4:**
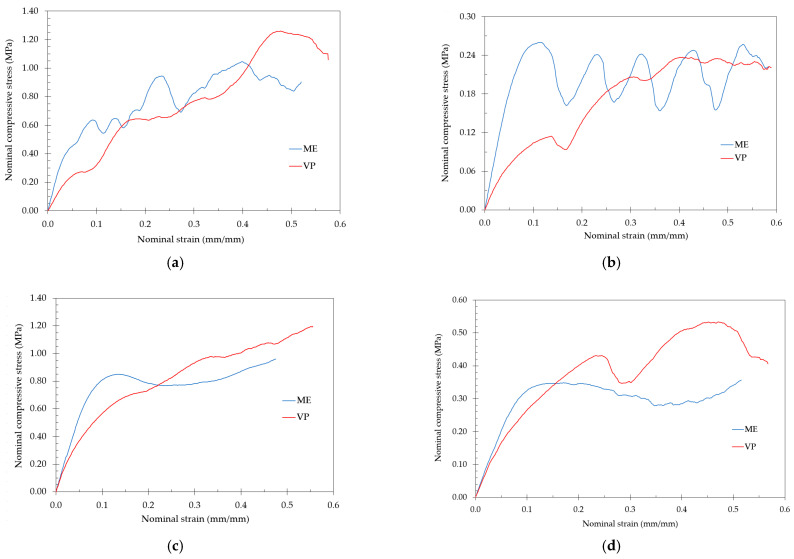
Stress-strain diagram for the lattices with the highest compressive strength: (**a**) TOV; (**b**) CD; (**c**) CF; and (**d**) HPL.

Figure 4 shows the stress-strain curves for the lattice structures fabricated by ME and VP with the highest strength during the compression test: TOV, CF, HPL and CD lattices. The stress-strain curves of ME-fabricated samples showed the typical plateau stress beginning at a nominal strain of about 0.1, with TOV and CD lattices presenting significant oscillations. On the other hand, in VP-fabricated lattices no plateau stress can be easily identified.

**Figure 5:**
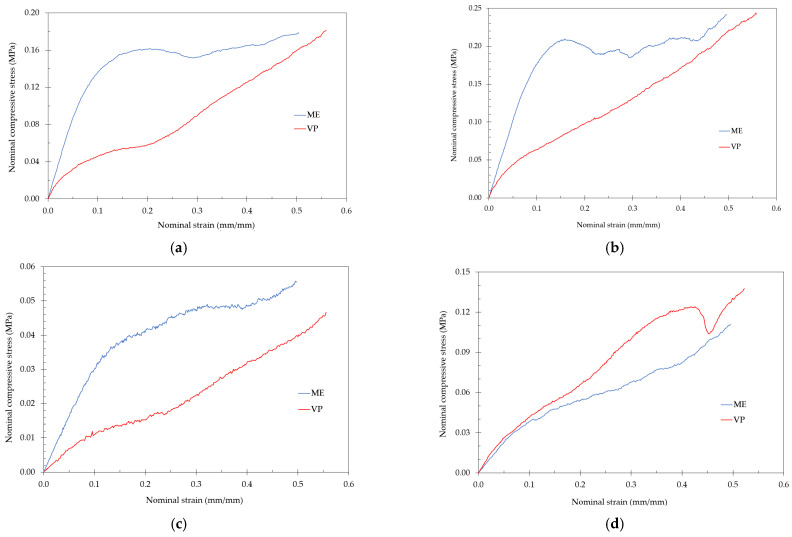
Stress-strain diagram for the lattices with the lowest compressive strength: (**a**) CVC; (**b**) TV; (**c**) HPV; and (**d**) HPD.

Figure 5 shows the stress-strain curves for the lattice structures fabricated by ME and VP with the lowest strength during the compression test: TV, CVC, HPD and HPV lattices. It is not possible to identify the plateau stress of the VP-fabricated lattices, with all of them showing a steady increase in compressive stress as the test progressed.

**Figure 6:**
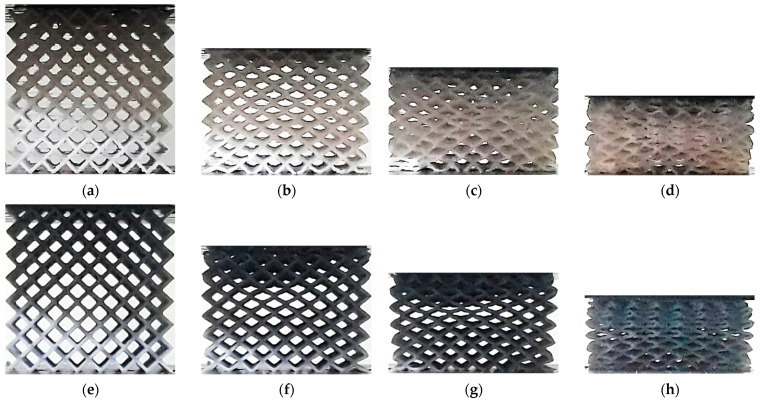
Deformation process in CVC lattice structures: (**a**) ME 0%; (**b**) ME ~15%; (**c**) ME ~30%; (**d**) ME ~45%; (**e**) VP 0%; (**f**) VP ~15%; (**g**) VP ~30%; (**h**) VP ~45%.

Photographs of the samples were taken during the compression tests. The images were taken from a single side of the sample, at intervals of approximately 5% nominal strain, starting at 0%. Figure 6 shows a comparison of the deformation process in CVC structures fabricated using ME and VP. No clear differences are noticeable in the early stages of the compression tests, up to a nominal strain of 15%, with no visible strain localization in either lattice. When the nominal strain reaches 30%, strain is still uniform inside the VP lattice, but some strain localization is visible in the diagonal of the ME lattice. A larger deviation is visible for a nominal strain of 45%, with uniformly distributed compacted cells in the upper region of the VP specimen, and more uneven deformation in the ME lattice, with compaction localized in the mid-section.

**Figure 7:**
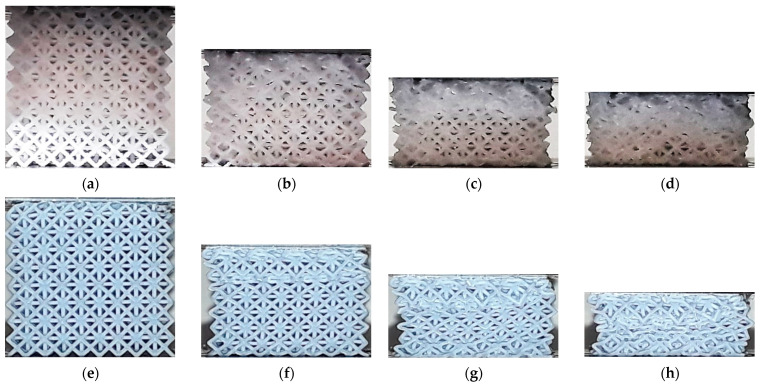
Deformation process in TOV lattice structures: (**a**) ME 0%; (**b**) ME ~20%; (**c**) ME ~35%; (**d**) ME ~50%; (**e**) VP 0%; (**f**) VP ~20%; (**g**) VP ~35%; (**h**) VP ~50%.

Figure 7 shows the deformation process in TOV lattices fabricated using ME and VP. At a nominal strain of 20% localization is visible in the upper and lower side of both specimens, but some differences are noticeable. The VP specimen shows the complete collapse of the upper first and third horizontal layers, in addition to the compaction of the layer on the lower side. On the other hand, in the ME specimen the compaction is unevenly distributed, with cell damage visible also on the left side.

When the nominal strain reaches 35%, cell collapse inside the VP lattice is localized in the upper and lower horizontal layers, although bent struts are visible in all layers. In the ME specimen, an undeformed core is visible in the lower half, with uneven local strain in the upper half. At a nominal strain of 50%, both specimens have almost fully collapsed structures, with a layer-by-layer pattern in the case of the VP lattice, and a more disorderly cell arrangement in the ME.

**Figure 8:**
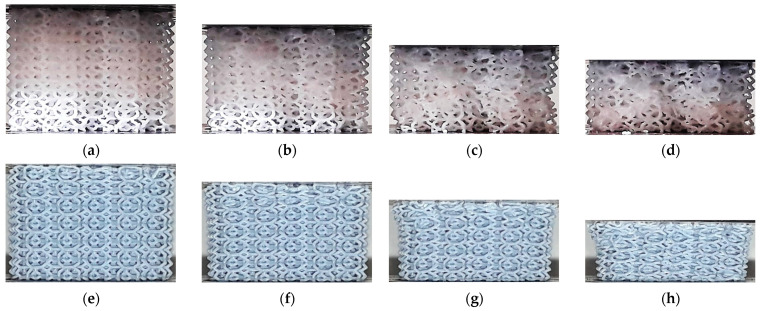
Deformation process in HPL lattice structures: (**a**) ME ~15%; (**b**) ME ~22,5%; (**c**) ME ~35%; (**d**) ME ~50%; (**e**) VP ~15%; (**f**) VP ~22,5%; (**g**) VP ~35%; (**h**) VP ~50%.

Figure 8 shows the deformation in HPL structures fabricated by ME and VP. No clear difference is evident in the early stages of the compression test, with strain localized in the upper layer, up to a nominal strain of 15%. However, when the nominal strain reaches around 25%, the onset of a diagonal band is visible in the ME specimen, while in the VP lattice the compression remains predominantly layerwise, with strain localization visible in the upper layer. The subsequent stages follow the same trend, with the VP lattice showing layer-by-layer collapse, and non-uniform deformation in the ME specimen.

**Figure 9:**
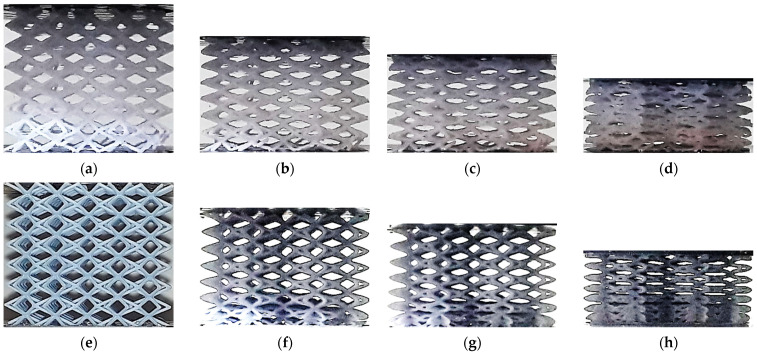
Deformation process in HPV lattice structures: (**a**) ME 0%; (**b**) ME ~20%; (**c**) ME ~30%; (**d**) ME ~45%; (**e**) VP 0%; (**f**) VP ~20%; (**g**) VP ~30%; (**h**) VP ~45%.

Figure 9 shows the compressive response of the HPV specimens fabricated. No significant difference is noticeable during the process between the two specimens. In contrast to other lattices, no strain localization is evident, both VP and ME lattices showing uniformly distributed compression across the lattice up to 45% nominal strain.

**Figure 10:**
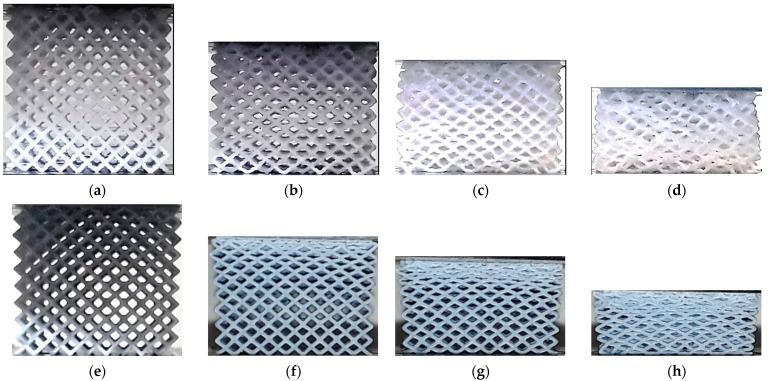
Deformation process in TV lattice structures: (**a**) ME 0%; (**b**) ME ~17%; (**c**) ME ~32.5%; (**d**) ME ~50%; (**e**) VP 0%; (**f**) VP ~17%; (**g**) VP ~325%; (**h**) VP ~50%.

Figure 10 shows the response of the TV specimens. The compaction process is different from the start, with visible strain localization in the upper layer of the VP specimen, in opposition to a uniformly distributed strain in the ME lattice. As the nominal strain increases the behavior remains the same in both specimens. In the VP sample, the collapse of the structure takes place on a layer-by-layer basis, just like in the other VP lattices, while the final collapse of cells in the ME specimen is disorderly.

**Figure 11:**
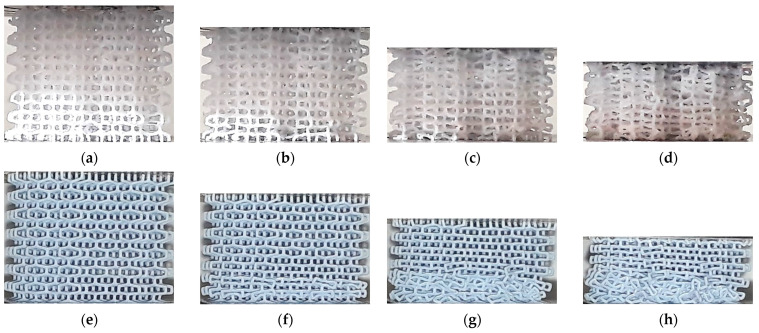
Deformation process in HPD lattice structures: (**a**) ME 0%; (**b**) ME ~12.5%; (**c**) ME ~30%; (**d**) ME ~40%; (**e**) VP 0%; (**f**) VP ~12.5%; (**g**) VP ~30%; (**h**) VP ~40%.

Figure 11 shows the evolution of the HPD specimens during the compression test. Similar to the TV lattices, strain in the VP specimen is localized in the upper layer, while a more uniformly distributed compaction is observed in the ME lattice. In the VP sample, the collapse of the structure takes place on a layer-by-layer basis, just like in the other VP lattices, while the final collapse of cells in the ME specimen is disorderly.

**Figure 12:**
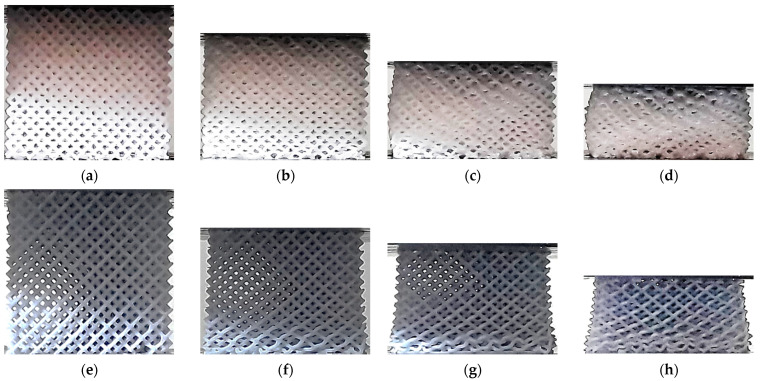
Deformation process in CF lattice structures: (**a**) ME 0%; (**b**) ME ~15%; (**c**) ME ~30%; (**d**) ME ~45%; (**e**) VP 0%; (**f**) VP ~15%; (**g**) VP ~30%; (**h**) VP ~45%.

Figure 12 shows the compaction of CF specimens under compression. Unlike other lattices, specimens’ cross-section expanded unevenly: barreling is visible in ME lattices, while in VP lattices the expansion occurred in the lower end. While localization is noticeable in the lower end of the VP specimen, deformation was uniform in the ME lattice.

**Figure 13:**
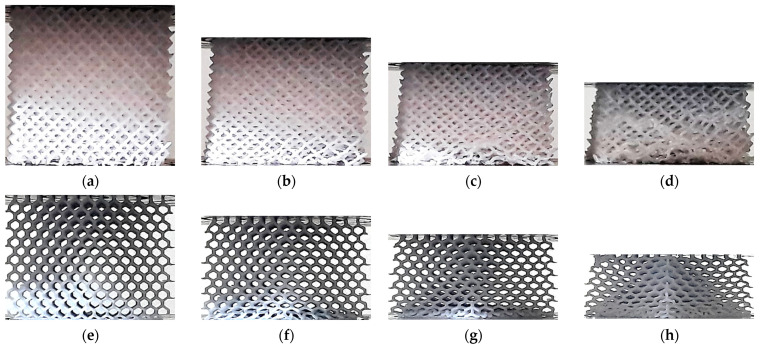
Deformation process in CD lattice structures: (**a**) ME 0%; (**b**) ME ~12.5%; (**c**) ME ~30%; (**d**) ME ~40%; (**e**) VP 0%; (**f**) VP ~12.5%; (**g**) VP ~30%; (**h**) VP ~40%.

Figure 13 shows the response of the CD specimens fabricated using ME and VP under compression from two different points of view. In both lattices, the compaction occurred in the same way, as the structure collapsed along a preferred inclined direction. The lower cross-section expanded and the cells in the deformed wedge-shaped region (Figure 13h) fractured. In the ME lattice, the deformation led to the partial fracture of the specimen along the plane, while in the VP specimen damage was present in all cells located in the wedge-shaped region.

Mean values and standard deviations for the elastic modulus and energy absorption capacity of the eight lattice configurations built by ME and VP are presented in Table 5 and Table 6. The energy absorption capacity quantified was measured as the area under the stress-strain curve up to a value of 40% nominal strain. No comparison of plateau stress was possible, as the compressive stress of VP specimens did not reach a plateau.

## 4. Discussion

**Figure 14:**
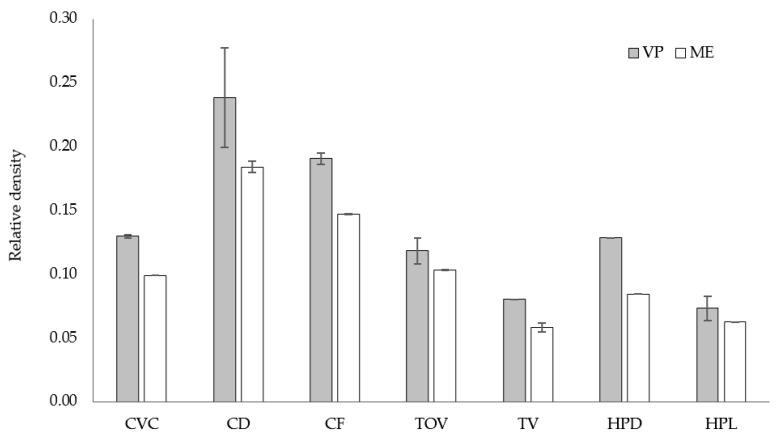
Relative density for the eight lattice structures built by ME and VP.

**Figure 15:**
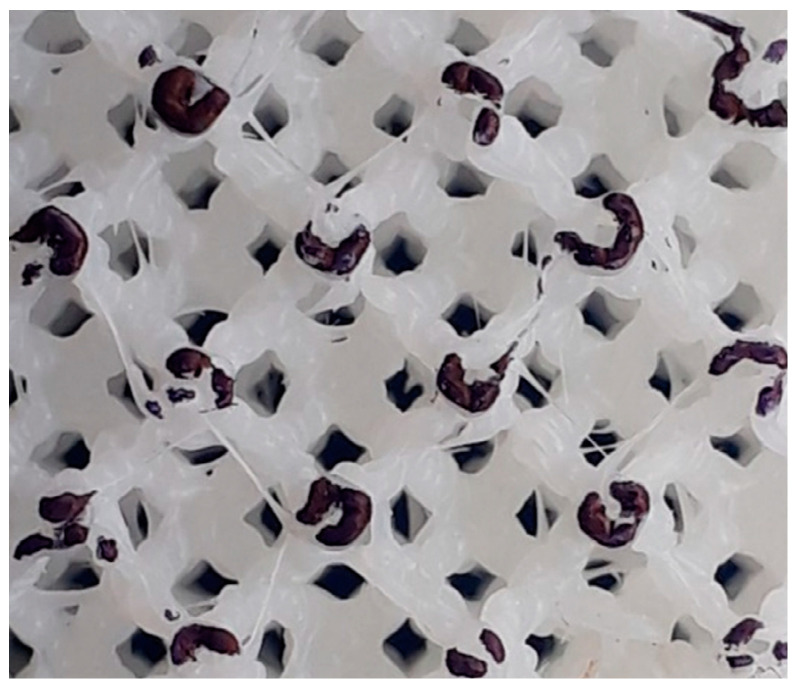
Cross-section of a CF lattice manufactured by ME. The colored surface highlights the deposed filament in a layer, with air gaps visible as the non-colored portion inside the darker contours.

Figure 14 shows a comparison of the relative density for all lattice structures fabricated by ME and VP, where the relative density is defined as the ratio between the density of the sample and that of the solid of which it is made [49]. Although the strut diameter of lattice structures fabricated by ME was larger, the relative density was smaller in all cases. This could be a consequence of the discontinuities (air gaps) inside the struts built by ME: the filament is deposed as a continuous string with air gaps left between them (Figure 15), thus causing a lower density in parts manufactured by ME.

The relative density ratio between lattices built by VP and ME was in the range of 1.24–1.52, somewhat higher than the 1.20 density ratio between VP and ME-fabricated solid samples (Table 3). The wider range could be attributed to geometric differences between the as-fabricated ME samples and the digital model: curved and inclined surfaces are transformed into layers and a trajectory is defined for the extruder, resulting in slight geometric changes and the amount of deposed filament.

A large standard deviation is noticeable in CD, TOV and HPL lattices fabricated by VP. This is consistent with observations in previous studies [37], which suggested difficulties in the removal of liquid photopolymer after the process in geometrically complex parts. Although the VP-lattices were washed in isopropyl alcohol and were visually inspected to verify that no residues were left inside them, CD, TOV and HPL have the most intricate geometry among the different lattices, making it difficult to effectively verify that no residual photopolymer remained in the lattice core.

In clear contrast to the ME samples, no clear plateau stress was visible in the stress-strain curves for the different VP-fabricated samples, especially in low-strength lattices (Figure 5). Some oscillations are visible in the stress-strain curves of the stronger lattices fabricated by DLP (TV, CVC, HPD and HPV) (Figure 4), which could be related to the local collapse of struts inside the structure. Similar behavior was reported previously in SLA-fabricated octet-truss and graded body-centered- cubic lattices [19,20]. Although in previous studies this response was attributed to the brittle response of the lattices or the collapse of the thinner struts in the lower density region, the cause in our study is unclear, as the compressive response of the lattices was diverse: some samples showed considerable elasticity, while others fractured.

No clear relationship could be identified between the stress-strain curves and the deformation mechanisms observed in the lattices (Figure 6, Figure 7, Figure 8, Figure 9, Figure 10, Figure 11, Figure 12 and Figure 13). For instance, the compaction mechanism was very similar in CVC and HPV lattices built by both AM methods, but the stress-strain curves were markedly different depending on the AM method. Furthermore, lattices with similar stress-strain curves for both VP and ME (HPL and HPD, up to ~15% nominal strain, Figure 4d and Figure 5d) showed different compaction mechanisms (Figure 8 and Figure 11). These observations suggest that stress-strain curves are not determined by how the compaction of cells occurs, but by other factors such as unit-cell type, relative density and the mechanical behavior of the solid material.

In VP lattices two compaction forms were observed: layer-by-layer densification, beginning on the upper or lower end of the specimens (CF, TOV, TV, HPL and HPD), and uniform compaction across all layers and cells (CVC and HPV). In the first case, the deformation process is defined by the relatively low strength of the struts, as the steeper struts in these lattices bent under compression and were unable to transmit the compressive load to other regions until the layer was fully compacted. On the other hand, in CVC and HPV lattices, the deformation was uniformly distributed, with the nodes acting as hinges that enabled the elastic compression of cells.

In ME specimens, a uniformly distributed compaction was reported in most lattices (CVC, TV, HPV, HPD and HPL). In contrast, TOV lattices showed layer-by-layer compaction, and barreling was observed in CF specimens in the final stage of the compression test (Figure 12d). However, in all cases, the compaction eventually evolved into uneven densification across multiple layers.

CD lattices built by both VP and ME showed a different compaction mechanism, as the collapse took place by the compaction of successive layers with a preferential orientation (Figure 13). The cells collapsed and the struts broke, leading to the fracture of the lattice along the preferential planes in both specimens. This highlights the importance of unit-cell type over solid material in CD lattices. The failure mechanism that leads to large oscillations in the stress-strain curve of some ME lattices could not be identified in the images captured during the process, revealing the shortcomings of the experimental setup. Nevertheless, a previous study remarked that these oscillations are spaced evenly, coinciding approximately with the cell size of the lattice [17]. The absence of such oscillations in the VP-fabricated sample suggests that the emergence of this phenomenon depends on both the unit-cell type and the mechanical properties of the solid material.

Partial elastic recovery was present in some lattices (up to 80% of their original height) after the load was removed, as previously reported for SLA lattices [18]. This behavior was observed in both ME and VP samples, and it is determined by the unit cell type. While in some lattices struts fractured under stress (CD, TOV, HPL) or were deformed permanently (CF), in some other configurations a large percentage of struts bent without suffering permanent damage (CVC, HPD), with the nodes acting as hinges. As the deformed beams pivoted backward on their hinges, the lattice recovered elastically.

Nevertheless, in some VP-fabricated lattices, the response varied between samples. While sample 1 of VP-fabricated lattices TV and HPV presented an elastic response, sample 2 behaved as a brittle material. The changes could be attributed to the degradation of the photocurable resin during the fabrication [1], in addition to the degradation of the PDMS coating that protects the digital light projector, which could cause a severe decrease in print quality [35].

The elastic modulus of ME-fabricated lattices was superior to that of VP-fabricated counterparts (Table 5), even when the VP-fabricated lattices had larger relative densities. The single exception was the HPD lattice, although the average elastic modulus of the VP lattice is within one SD of the mean value for the ME sample.

The largest elastic modulus values were obtained for the lattices with the highest density CF and TOV. On the other hand, the lowest values of elastic modulus were obtained for structures with the lowest density, lattices HPV and HPD. This outcome is consistent with the relationships proposed in the literature [49,50]. However, as both the mechanical properties of the solid material and the as-fabricated cell geometry differ between VP and ME samples, a direct comparison is difficult. To take into consideration these differences, the relative elastic modulus used in previous studies was calculated [21]. It is defined as the ratio between the elastic modulus of the lattice and that of the solid of which it is made for all materials [50].

**Figure 16:**
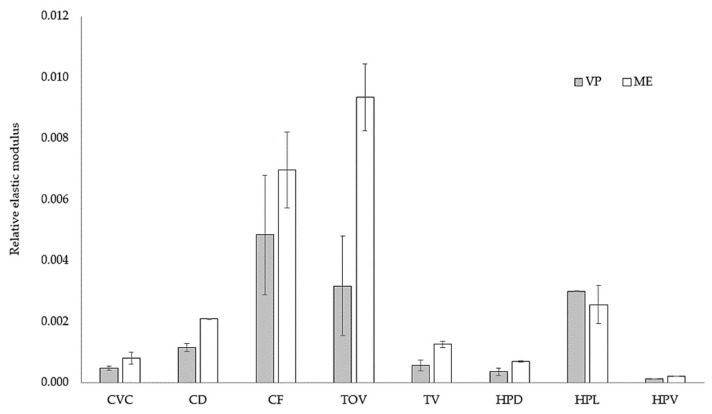
Relative elastic moduli for the lattice structures built by ME and VP.

Figure 16 presents a comparison of the relative elastic modulus for ME- and VP-fabricated lattices. Although the largest relative density and better overall accuracy of the VP-fabricated should lead to better mechanical properties, lattices fabricated by ME have better elastic moduli, with the HPL lattice as the only exception. Differences between ABS and VP resin should be canceled out by using the lattice-to-solid ratios, leaving only geometric parameters as relevant factors that could explain the gap between the stiffness in VP and ME samples: thicker struts in ME lattices leads to stiffer lattices, which is consistent with earlier studies [16,17].

The effect of the unit-cell type is also noticeable. For instance, the relative gap between TOV lattices is significantly larger than the difference between CF samples, and the relationship is inverted in HPL lattices. The link between relative elastic modulus and unit-cell type already reported by Luxner et al. [21] is valid when considering a wider variety of unit-cell types.

The differences between unit-cell types are not limited to their geometry, but could also be traced back to the fabrication routes in each AM process. Although the 3D digital model (STL) is the same, the pre-processing and manufacturing stages are different. During the pre-processing of the STL file, in DLP the model is pixelized, while in ME the trajectory of the extruder is defined, both altering the original geometry. Figure 15 shows evidence of the extruder’s path, reflected in the shape of the deposed filament. In the fabrication stage, part generation is controlled by the photopolymerization process in DLP, while in ME the molten filament is extruded through a heated nozzle, resulting in non-uniform struts [10], sagging and variations in the filament cross-section [51]. Although the measured diameter in ME lattices was larger, the cross-section is not uniform and is dependent on strut orientation, leading to variation in the properties of struts according to the unit-cell type.

A large standard deviation is noticeable for TOV and CF lattices fabricated by both VP and ME. As mentioned earlier, these lattices have the most intricate geometry among the benchmark structures and are in consequence the most challenging for the AM processes under consideration.

**Figure 17:**
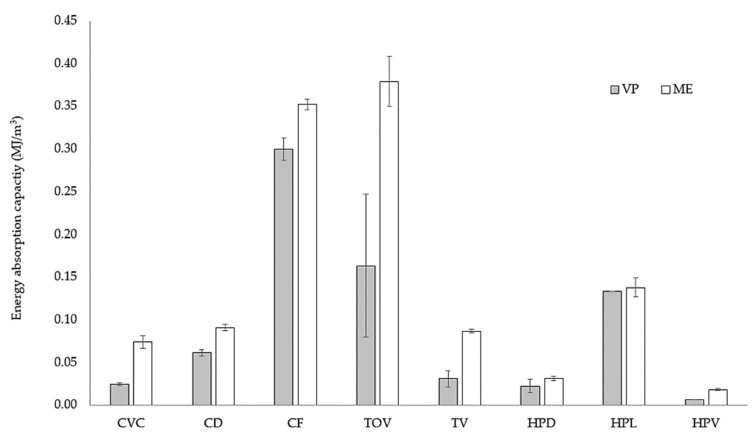
Energy absorption capacity up to 40% of the nominal strain for the lattice structures built by ME and VP.

Figure 17 shows a comparison of the energy absorption capacity for ME- and VP-fabricated lattices. The largest energy absorption was obtained with ME-fabricated lattices. Although both solid materials (ABS and resin) showed similar mechanical properties under compression, the response of the VP-fabricated lattice did not show the plateau stress typical of cellular materials. The effect of the unit-cell type is also noticeable in the energy absorption capacity of the lattices, with some lattices showing large discrepancies between ME and VP lattices (CVC, TV), while others had minimal differences (HPL). A large standard deviation is noticeable for the TOV lattice fabricated by VP. This could be attributed to a decreased in the quality of the second tested sample, possibly caused by the degradation of both the photocurable resin during [1] and the PDMS coating that protects the light projector during the fabrication [35].

Another important factor that could lead to differences in compression behavior between ME and VP samples is the bonding strength between layers of the feedstock material. While bonding strength is dependent on processing conditions [52], AM parameters were set according to the manufacturers’ guidelines and their influence on the mechanical properties was not explored.

## 5. Conclusions

Both AM methods—material extrusion and vat photopolymerization—were capable of producing the group of miniature lattice structures selected as a benchmark in this study.

The accuracy, quality and relative density of the VP-fabricated lattices were superior to that of their ME counterparts, although the latter showed predominantly higher elastic moduli and energy absorption capacity, even when the difference in feedstock material and relative density were taken into consideration. This could be related to several causes, including shape or size deviations introduced during the ME process or the degradation of the resin during the VP process.

Significant differences between samples produced by the two AM methods were also evident in the stress-strain curves: the VP samples did not show the plateau stress typical in cellular materials, in opposition to the ME lattices, suggesting that the mechanical properties of the solid material could be more important than the geometrical accuracy of the lattice. Thus, the limited variety of photocurable resins available for DLP could be a restriction, although recent developments in photopolymerizable resins and additives that enable composite materials could lead to improvements in the field [19,53,54]. ME-fabricated lattices could be a better option given the broader range of available materials, but their poor accuracy and low productivity must be considered.

No clear relationship could be identified between the stress-strain curves and the deformation mechanisms observed in the photographs taken during the compression tests, revealing the shortcomings of the experimental setup. Potential improvements in future studies might be achieved by improving the recording techniques, like high-speed and/or high-resolution photography. Nevertheless, denser lattices might require alternative methods to study the deformation process inside them.

## Figures and Tables

**Table 1:** Nominal geometric features of tested unit cells and lattice structures [17].

		Lattice Dimensions			Struts			
	Unit Cell Type	Height (mm)	Width (mm)	Length (mm)	Diameter (mm)	Length (mm)	Overhang Angle (°)	Cell Size (mm)
CVC	Cube vertex centroid	31	31	31	0.70	4	45	5
CD	Cubic diamond	34	34	34	0.70	2.5	65	3
CF	Cubic fluorid	33	33	33	0.70	2.5	65	4
TV	Tet vertex centroid	30	30	30	0.70	3	55	5.5
HPV	Hex prism vertex	34	34	34	0.70	5	60	4.5/6.5
TOV	Tet oct vertex centroid	31	31	31	0.70	3/3/4.5	0/55/90	4.5/6.5
HPD	Hex prism diamond	34	34	39	0.70	3/2	65/90	3/5
HPL	Hex prism laves phase	34	34	39	0.70	3/2	0/60	4.5/5.5

**Table 2:** Mechanical properties of ABS and resin reported by the manufacturers.

Feedstock Material	ABS [44,45]	VP [46]
Density (g/cm^3^)	1.05	1.05–1.25
Elastic modulus (MPa)	2180–2230	1779–2385
Tensile strength (MPa)	26–31	30–52

**Table 3:** Mechanical properties of ABS and photopolymerized resin.

Feedstock Material	ABS	VP Resin
Density (g/cm^3^)	0.98–1.01	1.19–1.20
Elastic modulus (MPa)	1230–1512	1118–1376
Compressive yield stress (MPa)	36.9–44.4	25.9–27.3

**Table 4:** Mean values and standard deviation of density for the lattice structures specimens.

Unit Cell Type	Density (g/cm^3^)			
	Material Extrusion		Vat Photopolymerization	
	Mean	SD	Mean	SD
CVC	0.1030	7.07 × 10^−4^	0.1416	3.37 × 10^−3^
CD	0.0971	7.07 × 10^−5^	0.1495	1.25 × 10^−3^
CF	0.1805	4.31 × 10^−3^	0.2750	4.50 × 10^−2^
TV	0.1013	3.54 × 10^−4^	0.1364	5.15 × 10^−3^
HPV	0.0612	2.12 × 10^−4^	0.0845	1.16 × 10^−2^
TOV	0.1442	4.24 × 10^−4^	0.2196	1.63 × 10^−4^
HPD	0.0569	3.54 × 10^−3^	0.0924	3.65 × 10^−4^
HPL	0.0828	7.07 × 10^−5^	0.1483	1.09 × 10^−2^

**Table 5:** Mean values and standard deviation of elastic modulus for lattice structures.

	Elastic Modulus (MPa)			
	Material Extrusion		Vat Photopolymerization	
	Mean	SD	Mean	SD
CVC	1.07	2.67 × 10^−1^	0.60	8.05 × 10^−2^
CD	2.80	2.26 × 10^−2^	1.44	1.71 × 10^−1^
CF	9.36	1.67 × 10 ^0^	6.02	2.43 × 10 ^0^
TV	1.69	1.34 × 10^−1^	0.70	2.23 × 10^−1^
HPV	0.30	3.46 × 10^−3^	0.15	6.36 × 10^−4^
TOV	12.55	1.46 × 10 ^0^	3.94	2.02 × 10 ^0^
HPD	0.93	2.79 × 10^−2^	0.44	1.49 × 10^−1^
HPL	3.44	8.34 × 10^−1^	3.72	1.27 × 10^−2^

**Table 6:** Mean values and standard deviation of energy absorption capacity for lattice structures.

	Energy Capacity (MJ/m^3^)			
	Material Extrusion		Vat Photopolymerization	
	Mean	SD	Mean	SD
CVC	0.0738	7.33 × 10^−3^	0.0377	6.20 × 10^−3^
CD	0.0908	3.81 × 10^−3^	0.0889	9.91 × 10^−3^
CF	0.3521	5.92 × 10^−3^	0.4068	4.38 × 10^−2^
TV	0.0865	1.96 × 10^−3^	0.0460	1.55 × 10^−2^
HPV	0.0182	1.36 × 10^−3^	0.0099	0.00 × 10 ^0^
TOV	0.3790	2.93 × 10^−2^	0.2455	1.29 × 10^−1^
HPD	0.0313	2.69 × 10^−3^	0.0318	1.12 × 10^−2^
HPL	0.1377	1.12 × 10^−2^	0.1853	0.00 × 10 ^0^

## Data Availability

Data available on request.
